# Magnetic Fields Disperse *Escherichia coli* from Biofilm via CsrA and RpoE Regulation

**DOI:** 10.3390/microorganisms14102200

**Published:** 2026-10-01

**Authors:** Pengbo Wang, Xiaoxiao Liu, Shuhan Dai, Yunchong Li, Min Yang, Yunjun Yan

**Affiliations:** Key Laboratory of Molecular Biophysics of the Ministry of Education, College of Life Science and Technology, Huazhong University of Science and Technology, Wuhan 430074, China; wangpb@hust.edu.cn (P.W.);

**Keywords:** biofilm, magnetic field treatment, *E. coli*, metabolism regulation, periplasmic stress

## Abstract

The biofilm formation of pathogenic bacteria improves their infectivity, virulence, and stress tolerance. Therefore, inhibition of biofilm is beneficial for antibiosis. Magnetic fields (MFs) have been found to suppress the adhesion of *Escherichia coli*, but their underlying molecular mechanisms remain unclear. This study aims to explore the effects of different MFs on *E. coli* biofilm and the detailed molecular mechanism. The quantitative results of crystal violet staining showed that the biofilm significantly decreased by 20.5% (±6.3%) with 2.5 T pulse MF (PMF) treatment, 24.7% (±13.2%) with 15 mT rotating MF (RMF) treatment, and 14.9% (±8.8%) with 100 mT static MF (SMF) treatment. No obvious cell death or change in cellular metabolism was observed after 1 h RMF treatment, while the number of planktonic cells increased, indicating that 1 h RMF treatment dispersed biofilm without killing cells. Transcriptome profiling of PMF-treated *E. coli* revealed significant downregulation of core genes associated with biofilm formation, such as *pgaA* and *csgD*. This was associated with the upregulation of the gene *csrA* and the activation of transcription factor RpoE, which inhibits the translation of *pgaA* and *csgD,* respectively. qRT-PCR analysis and promoter activity assay verified these molecular changes in biofilm-forming cells under PMF and RMF. The essential roles of CsrA and RpoE in RMF-mediated biofilm inhibition were further supported by gene overexpression, knockout, or silencing. Additionally, it was demonstrated that RMF exerted consistent anti-biofilm effects in *E. coli* O157:H7 and *Bacillus subtilis* WB800N. Thus, the findings of this study provide evidence for the molecular mechanism of MF on *E. coli* biofilm inhibition.

## 1. Introduction

Biofilms are complex structures formed by microbial cells and extracellular polymeric substances (EPS) primarily composed of polysaccharides, proteins, and DNA [1]. They enable microorganisms to adhere to the surfaces of various materials, enhance their environmental adaptability [2], and improve the infectivity of many pathogenic bacteria [3]. For instance, *E. coli* O157:H7 can adhere to food and various equipment via biofilm, causing serious health issues [4,5]. Uropathogenic multidrug-resistant *E. coli* also forms robust biofilms contributing to gynecological and reproductive-related infections [6]. Biofilms also enhance bacterial resistance to antimicrobial agents [7,8]. The barrier formed by EPS and persister cells in biofilms reduces the sensitivity of bacteria to antimicrobials [9], which complicates the treatment of infections and renders them prone to recurrence [10,11]. Furthermore, the formation of biofilms is often accompanied by the production of many toxins, increasing their harmfulness [11,12]. Therefore, the removal of biofilms is an important issue in the treatment of bacterial infectious diseases [7,13].

To remove biofilm, high concentrations and prolonged use of antibiotics are usually required, increasing the risk of antibiotic resistance [14]. Thus, multiple new methods are being developed to mitigate this risk, such as photodynamic therapy [15], phage-based therapy [16,17] and antibacterial material [18]. Magnetic field (MF), which is a common physical stimulus that has been applied in medical fields such as wound healing [19,20] and inflammation reduction [21], has been reported to affect microbial biofilm. The adhesion of *E. coli* is reduced by 500 mT SMF treatment [22]. The biofilm of *Helicobacter pylori* diminishes after 50 Hz and 1 mT sinusoidal MF treatment for 2 days, with an increase in the proportion of bacterial cell death [23]. However, the biofilm of *Enterococcus faecalis* increases after 170 mT SMF treatment [24], and the same was true for *Pseudomonas aeruginosa* treated with 200 mT SMF [25]. Many studies have reported MF antibacterial effects, possibly related to biofilm responses. The diameter of *E. coli* colony was reduced by 250 mT SMF treatment via impaired carbon source utilization and long-chain fatty acid degradation, while the number of viable CFUs remained unchanged [26]. Treatment with 450 mT SMF decreases the survival of *E. coli*, and the effect exhibits greater significance as exposure duration increases [27]. However, 300 mT SMF treatment significantly accelerates the growth of *E. coli* cultured in LB medium supplemented with glutamate [28]. The MF antibacterial effects seem to depend on some other conditions, such as exposure time, MF strength, and culture conditions. Unraveling the mechanisms of MF effects on microorganisms under defined conditions helps to explain these conflicting results. In addition, owing to its safety and strong penetrability to organisms [21], MF technology holds promising application prospects for the mitigation of biofilms formed on fresh foods and invasive medical devices. The inconsistent responses of different microorganisms to MF restrict the development of this technology. Elucidating the precise molecular mechanisms of MF-biofilm interactions is therefore essential to break through current limitations.

The formation and maintenance of *E. coli* biofilm depend on EPS synthesis and low motility. The EPS of *E. coli* biofilm mainly consists of water-soluble polysaccharides, proteins, extracellular DNA, and water-insoluble compounds [29]. Polysaccharides are the matrix component of biofilm and have three main types in *E. coli*, including poly-β-1,6-N-acetyl-D-glucosamine (PGA) synthesized by the *pgaABCD* operon, cellulose synthesized by the *yhjR-bcsQABZC* operon and *bcsEFG* operon, and colanic acid (CA) synthesized by the *wca* gene cluster [9]. Curli fimbriae account for up to 85% of the biomass of *E. coli* biofilm and are encoded by the *csgBAC* operon and the *csgDEFG* operon [30]. The motility of *E. coli* relies on flagella, which are regulated by the *flhDC* operon and the FlhDC regulon [31]. These genes are the main effectors associated with *E. coli* biofilm.

Some regulatory systems affect the transition of *E. coli* between the planktonic and biofilm states by regulating the expression of these effector genes. For instance, 3′,5′-cyclic diguanylic acid (c-di-GMP), which is a second messenger synthesized under various environmental stimuli, promotes the synthesis of curli, cellulose, and PGA, and inhibits flagellar synthesis, thus promoting biofilm formation [32]. Quorum sensing systems affect EPS synthesis, flagellar motility, and biofilm [33,34]. Stress-associated transcription factors RpoS and RpoE promote and inhibit biofilm formation by regulating related gene transcription, respectively [35]. Partial two-component signaling systems are also involved in biofilm formation [36]. In addition, some metabolism-related regulators, such as CsrA, also regulate biofilm by affecting EPS synthesis [37]. Through these regulatory systems, *E. coli* senses its internal and external conditions and regulates biofilm formation. These systems also serve as targets for biofilm elimination methods [34,38]. However, it remains unknown which system MF acts on to reduce *E. coli* biofilms.

This study aims to explore the mechanism by which MF affects *E. coli* biofilm. The effects of different MF treatments on *E. coli* biofilm were evaluated first. High-intensity pulsed MF (PMF), rotating MF (RMF), and static MF (SMF) were used. Cell viability and metabolic activity were measured after RMF treatment. Then, transcriptome sequencing and analysis of planktonic *E. coli* cells treated with PMF were conducted. qRT-PCR was conducted to verify the related gene expression changes in biofilm cells. Overexpression, knockout, or activity inhibition of these genes were performed to further evaluate their roles in the biofilm reduction caused by MF. Furthermore, the effects of RMF on biofilms of multiple bacterial species were measured.

## 2. Materials and Methods

### 2.1. Strain Preparation

Strains used from Section 3.1, Section 3.2, Section 3.3, Section 3.4 and Section 3.5 were derived from *E. coli* BL21(DE3), which is a commercial strain purchased from Shanghai Weidi Biotechnology Company (Shanghai, China). *E. coli* O157:H7 and *Bacillus subtilis* WB800N strains used in Section 3.6 are from laboratory storage. The operation of *E. coli* O157:H7 was conducted in a biosafety level-2 laboratory. Standard techniques were employed for cloning, electroporation, and other genetic manipulations. *E. coli* was cultured in Luria–Bertani (LB, 1% tryptone, 0.5% yeast extract, 1% NaCl) at 37 °C when constructing these strains. Strains and plasmids used are described in Supplementary Table S1. The primers used in these processes are listed in Supplementary Table S2. Genes were amplified from BL21(DE3) genomic DNA (GenBank: CP053602.1) via PCR. Genomic coordinates of the respective genes are listed in Supplementary Table S3.

For gene overexpression, including *rpoE*, *rseAB*, *csrA*, *csgD*, and *pgaABCD*, the pET-28a plasmid was used. For overexpression of *rybB* and *csrB*, which are sRNAs, the complete gene sequences with their natural promoters and terminators were cloned into pET-28a plasmids. The protein RseA is an anti-sigma factor of RpoE [39], and the sRNA CsrB is a small regulatory RNA of CsrA [40]. Overexpression of these genes serves as an alternative to gene knockout. It does not fully abolish CsrA or RpoE activity but only suppresses their function. Either SDS-PAGE or qRT-PCR was used to verify gene overexpression. The CRISPR/Cas9 technology was used to knock out *rybB*, *csgD*, *pgaA,* and *lacZ*. It was performed as previously reported [41]. Detailed experimental procedures are provided in Supplementary Methods. *E. coli* BL21(DE3) was used as the host strain for gene overexpression and knockout.

For determination of β-galactosidase activity, the reporter vectors regulated by RpoS and RpoE were constructed using the pET-28a plasmid as the backbone. The T7 promoters of pET-28a were replaced by the promoter of *sodC* in pET-P*sod* and by the promoter of *rpoH* in pET-P*rpoH*p3. The *lacZ* gene was inserted into the multiple cloning site using BamHI and NotI. The pET-P0-*lacZ* plasmid, whose multiple cloning site lacks any promoter but carries the *lacZ* gene, was used as the control plasmid. The three reporter vectors were transformed into the BL21-Δ*lacZ* strain. Detailed experimental procedures are provided in Supplementary Methods.

### 2.2. Biofilm Formation and Observation

Overnight cultures of different strains were diluted to an OD_600_ of 0.5 in fresh LB medium and loaded 150 μL per well into 96-well microtiter plates (polystyrene, Corning Incorporated, Corning, NY, USA). For gene-overexpressing strains, kanamycin (Solarbio, Beijing, China) at a final concentration of 50 μg/mL and IPTG at 1 mM were added simultaneously for induction. Untreated polystyrene is hydrophobic with a near-neutral surface charge, easily adhered to by negatively charged *E. coli* [42], and is the widely accepted standard substrate for quantitative bacterial biofilm crystal violet assays [43]. The plates were cultured without shaking for 48 h at 30 °C to form biofilm. The biomass of biofilm attached to the microtiter plate was estimated by crystal violet staining. After discarding the cell cultures, the plates were washed twice with 160 μL phosphate-buffered saline (PBS, pH 7.2). Then the cleaning solution was discarded as much as possible, and 170 μL 0.1% (*w*/*v*) crystal violet aqueous solution (Solarbio, Beijing, China) was added to each well. After 20 min incubation for staining, the plates were washed twice with 200 μL distilled water and then air-dried completely at room temperature. Next, 200 μL absolute ethanol was added to solubilize the stained crystal violet. OD_595_ was measured with a microplate reader (FlexStation3, Molecular Devices, Sunnyvale, CA, USA) and used as an estimate of biofilm biomass. The comparisons of biofilm were conducted between the groups treated with MFs and the control groups treated with the same environment and duration without MF exposure. At least five biological replicates were performed for each experiment.

For microscopic observation, biofilm was cultured on glass slides following the method of Priscyla dos Santos Ribeiro et al. [44]. Glass slides are hydrophilic with a negative surface charge and are unfavorable for initial adhesion of *E. coli* [42]. But they are suitable for high-resolution imaging. Clean glass slides were put into 50 mL centrifuge tubes and sterilized by autoclaving at 121 °C and 100 kPa for 20 min. Subsequently, 10 mL of overnight culture was diluted to an OD_600_ of 0.5 with fresh LB medium and added to the centrifuge tube with the glass slide. The tubes were incubated horizontally with glass slides kept perpendicular to the liquid surface. After incubation without shaking for 48 h at 30 °C, biofilms formed in the region approximately 1 cm from the lower edge of the slides, determined by the liquid level in the tube. After incubation, the tubes containing glass slides and the original culture medium were also placed horizontally on the device for RMF treatment. Therefore, biofilms formed on glass slides can be exposed to MFs in the same manner as those cultivated on 96-well microtiter plates. After MF treatment, the slides were washed twice with distilled water and stained with 0.1% (*w*/*v*) crystal violet aqueous solution. Control samples were treated in the same manner under identical conditions, except for the absence of MF treatment. After 20 min incubation for staining, the slides were washed twice with 200 μL distilled water and observed under a microscope (FV3000 with DP23, Olympus, Hachioji, Japan). ImageJ (1.54f) was used to quantify images by calculating the ratio of crystal violet-stained area to the total image area. Three biological replicates were performed.

### 2.3. Magnetic Fields Treatment

**Figure 1:**
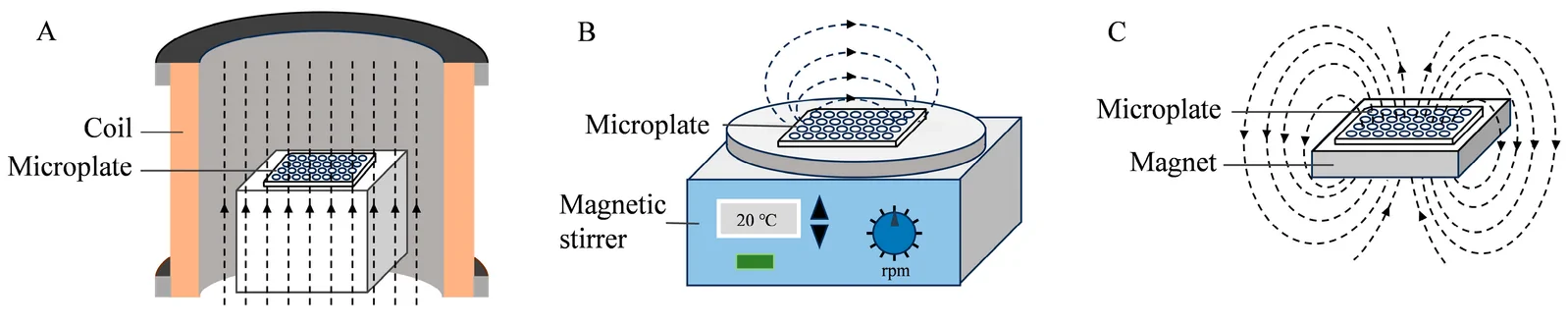
Schematic diagram of MF treatment equipment and MF near plates. (**A**). PMF treatment equipment diagram displaying the coil and sample plate. (**B**). RMF treatment equipment diagram displaying the magnetic stirrer and sample plate. (**C**). SMF treatment equipment diagram displaying the magnet and sample plate.

The plates and slides with cultivated biofilms were placed in various MFs in treatment groups. The control group of each treatment group was treated under identical conditions except for the MF. The MF settings were as follows. For PMF, fully charged capacitors discharge current into the treatment chamber, which is made of a coil to generate MF. The coil was 30 cm high with an inner diameter of 40 cm. Samples were placed at the center of the coil and at half the height. The intensity in the sample area was 2.5 T, and the pulse count was 15. There was a 5 min interval between every 3 pulses to maintain temperature stability. PMF treatment took a total of 1 h, including treatment time, interval time, and transportation time. The RMF was generated by a magnetic stirrer (Keer Instrument, Wuhan, China). The working area was a 15 cm-diameter circle. The MF originates at the bottom and decays rapidly toward the periphery. Samples were placed at the center of the working area to achieve maximal MF exposure. The average MF intensity across the entire sample area was approximately 15 mT. The rotational speed used was 50 Hz. The SMF was generated by a cuboidal NdFeB magnet (AIC MAGNETICS Ltd., Shanghai, China) with dimensions of 10 cm (length) × 5 cm (width) × 1.5 cm (height). The magnet was placed directly underneath the sample, and the average magnetic field intensity in the sample treatment area was 100 mT. The detailed spatial distribution of RMF and SMF intensity was measured using a gaussmeter (TUNKIA, Changsha, China) and shown in Supplementary Figure S1. The ambient temperature during treatment was maintained at approximately 27 °C by air conditioning and measured using an infrared thermometer (Chint, Shanghai, China). Experimental and control treatments were conducted simultaneously for each MF. The schematic diagram of the devices is shown in Figure 1.

For the 8 h long-term treatment, the temperature of the working area was measured every 30 min, as shown in Supplementary Figure S1C. During this long-term treatment, all samples, including control and experimental groups, were placed inside the treatment chamber for a total of 8 h. For RMF-treated groups, samples were loaded into the RMF sequentially from the longest exposure to the shortest. For example, samples for the 2 h treatment group were placed into the RMF at the 6th hour. All RMF-treated groups finished treatment simultaneously. Eight identical magnetic stirrers were used to ensure consistent RMF across all experimental groups.

### 2.4. Live/Dead Cell Staining

For live/dead cell staining, NucGreen and EthD-III (Uelandy, Suzhou, China) were used to stain live and dead cells, respectively. To prepare the working staining solution, 1 μL of NucGreen and 2 μL of EthD-III were mixed with 8 μL PBS. For planktonic cell staining, 1 μL of this working solution was added to 100 μL planktonic cell suspension, followed by 15 min of incubation in the dark before direct microscopic observation. For biofilm cells on glass slides, the working solution was further diluted 100-fold with PBS. Then, 15 μL of the diluted working solution was applied onto the biofilm, covered with a coverslip, incubated in the dark for 15 min, and then observed under a confocal fluorescence microscope (FV3000, Olympus Corporation, Hachioji, Japan). ImageJ was used to calculate the ratio of live cell counts to total cell counts for image quantification. Three biological replicates were performed.

### 2.5. MTT Viability Assay

MTT Cell Proliferation Assay Kit (Yessen Biotech Co., Ltd., Shanghai, China) was used. Following MF treatment, 10 μL of 5 mg/mL MTT working solution was directly added to 96-well plate wells (containing culture medium, planktonic and biofilm-attached cells). After gentle mixing, the plate was incubated at 37 °C for 10 min and centrifuged at 4000 rpm for 10 min. The supernatant was carefully removed, and 100 μL of formazan solubilization solution supplied with the MTT kit was added to each well, followed by immediate mixing. Samples were incubated at 37 °C for 4 h for complete dissolution, and absorbance was read at 570 nm. LB medium served as a blank control. After blank subtraction, data were compared with the untreated group.

### 2.6. Cell Counting

After MF treatment, the culture medium was gently pipetted to homogenize planktonic cells. The planktonic cells were then serially diluted with sterile PBS. An aliquot of 100 μL of the appropriately diluted cell suspension was spread onto LB agar plates. After incubation at 37 °C for 16 h, the number of colonies on the plates was counted.

### 2.7. RNA Extraction and Transcriptome Sequencing

*E. coli* was cultured in 50 mL LB medium for 19 h at 37 °C and 200 rpm. Then the culture was centrifuged to remove the supernatant and resuspended in an equal volume of PBS. After treatment with PMF at 2.5 T and 15 pulses, untreated and treated samples were collected for RNA extraction using the MolPure TRleasy Plus Total RNA Kit (Yessen Biotech Co., Ltd., Shanghai, China). NanoDrop 2000 spectrophotometer (Thermo Fisher Scientific, Waltham, MA, USA) was used to detect RNA quality and concentration. RNA integrity was evaluated using the Agilent 2100 Bioanalyzer (Agilent Technologies, Inc., Santa Clara, CA, USA) and gel electrophoresis. The libraries were constructed using TruSeq Stranded Total RNA with Ribo-Zero Gold (Illumina, Inc., San Diego, CA, USA). Then sequencing was conducted on HiSeqTM 2500 (Illumina, Inc., San Diego, CA, USA). Two biological replicates were performed.

### 2.8. Transcriptome Analysis

Fastp (v0.23.4) was used to filter raw reads, and STAR (v2.7.11a) was used to align clean reads to the reference genome of *E. coli* BL21 (DE3). The expression abundance of each gene was counted via sequence similarity comparison. The number of reads aligned to each gene was obtained, and gene counts were calculated using StringTie (v2.2.1). The gene counts were normalized by DESeq2 (v1.40), and *p*-values and fold changes (FC) of the difference comparison were calculated. The differentially expressed genes (DEGs) were screened based on false discovery rate (FDR) < 0.05 and FC > 2 and shown in Supplementary Materials S3. The Gene Ontology (GO) and Kyoto Encyclopedia of Genes and Genomes (KEGG) enrichment analysis of the differentially expressed genes were performed by clusterProfiler (v4.0) and also displayed in Supplementary Materials S3. The links for all software were listed in Supplementary Table S4.

### 2.9. Reverse Transcription Quantitative PCR (qRT-PCR)

Significantly, transcriptome sequencing was performed on shake-flask planktonic cells, instead of biofilm cells. Whether the same gene expression changes occurred in biofilm cells was unclear, making it critical to validate these expression changes in biofilm cells using qRT-PCR. *E. coli* was cultured in microplates according to the aforementioned method of biofilm formation. After MF treatment or sham treatment, cultures in the 96-well microtiter plate (Corning Incorporated, Corning, NY, USA) were vigorously and repeatedly pipetted to detach biofilm cells. Samples containing both planktonic and biofilm cells were collected, and total RNA was extracted using the MolPure TRleasy Plus Total RNA Kit (Yessen Biotech Co., Ltd., Shanghai, China). The reverse transcription was performed using Hifair II 1st Strand cDNA Synthesis SuperMix for qPCR (gDNA digester plus) (Yessen Biotech Co., Ltd., Shanghai, China). The RT-qPCR was processed using Hieff qPCR SYBR Green Master Mix (Low Rox Plus) (Yessen Biotech Co., Ltd., Shanghai, China) on a QuantStudio 3 (Thermo Fisher Scientific, Waltham, MA, USA). The operation processes were performed using standard techniques. The primer sequences used were in Supplementary Table S2. The *rpoD* gene was used as a housekeeping gene. Three biological replicates were performed.

### 2.10. Determination of β-Galactosidase Activity

The strains with reporter plasmids were cultured and treated with MFs according to the aforementioned method. Then 1 mL of culture from each group was taken as a sample. The samples were centrifuged, and the supernatant was discarded. The bacterial pellets were resuspended in 1 mL Z-buffer (60 mM Na_2_HPO_4_, 40 mM NaH_2_PO_4_, 10 mM KCl, 1 mM MgSO_4_ 7H_2_O, 0.1 M β-mercaptoethanol, pH = 7), and then OD_600_ of each sample was measured. Afterward, 50 μL 0.1% (*w*/*v*) SDS and 100 μL chloroform were added to each sample, and they were shaken for 15 s using the vortex mixer. After incubation at 30 °C for 5 min, 200 μL of 400 mg/mL ortho-nitrophenyl-β-galactoside (ONPG, Sigma, Beijing, China) was added to each sample, and they were further incubated at 30 °C for 5 min. Then 500 μL of 1 mol/L Na_2_CO_3_ was added to terminate the reaction. After centrifuging at 4 °C and 12,000 rpm for 3 min, the OD_420_ and OD_550_ of the supernatant were measured. β-galactosidase activity was expressed in Miller units (U) and calculated as follows:

$$Miller\;U=1000\times \left(OD_{420}-1.75\times OD_{550}\right)÷\;\left(T\times V\times OD_{600}\right)$$

*T* is the reaction time (5 min), and *V* is the sample volume (1 mL). The enzyme activity of the control strain LacZ-R was consistently determined to be zero; hence, these data were not displayed. To represent the changes in promoter activity that occur during treatment, the enzyme activities of each group were expressed as the enzyme activity after treatment minus the enzyme activity before treatment. Three biological replicates were performed.

### 2.11. Statistical Analysis

The experimental results were expressed as mean ± standard deviation. One-way analysis of variance (ANOVA) was performed for statistical comparisons, with significance set at *p* < 0.05, using SPSS 13.0 (SPSS Inc., Chicago, IL, USA; currently acquired by IBM). All graphs and calculations were performed using Origin 2025b (OriginLab Corporation, Northampton, MA, USA).

## 3. Results

### 3.1. Effects of Different MFs on E. coli Biofilm

**Figure 2:**
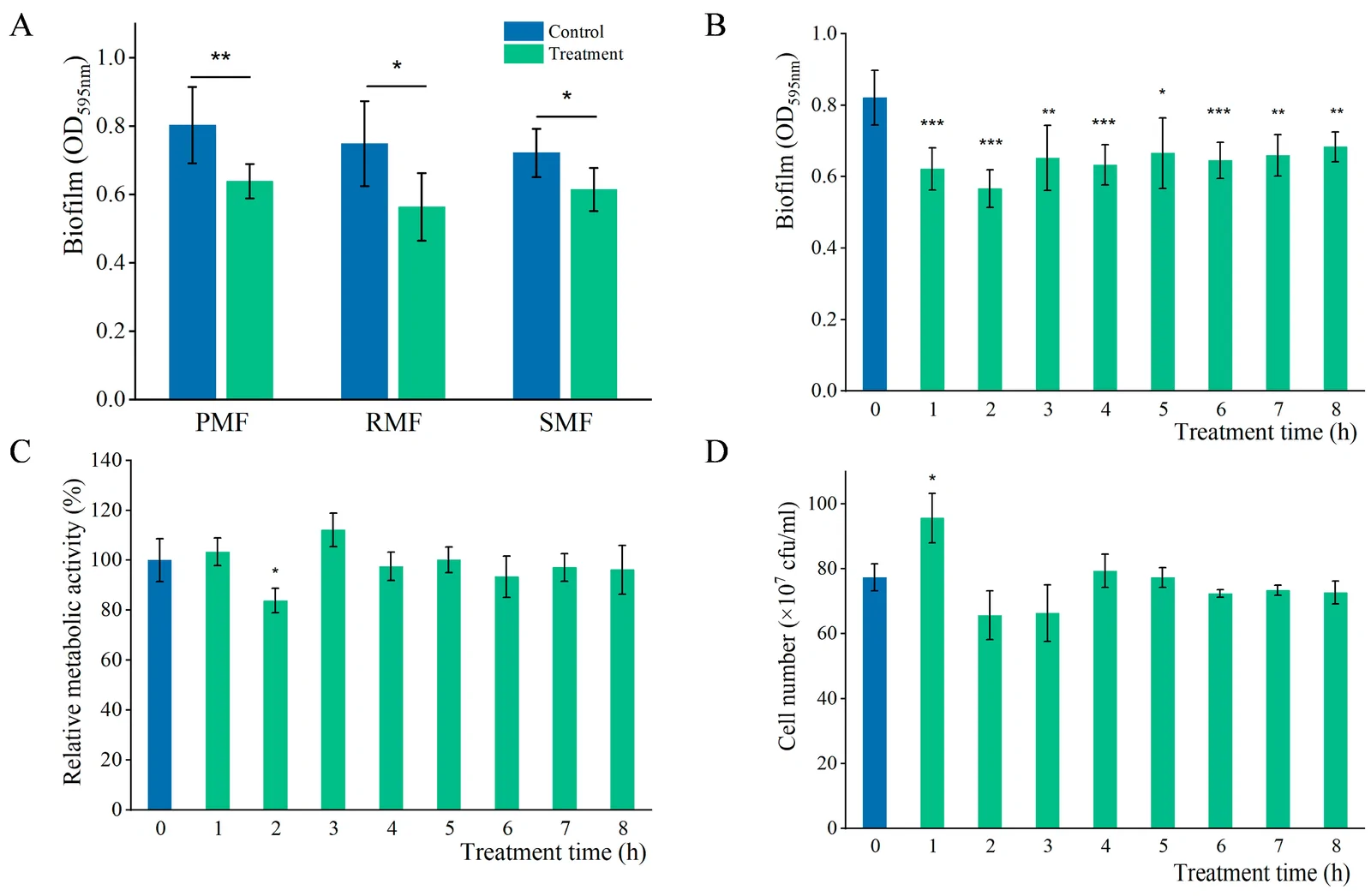
Effects of MFs on *E. coli* biofilm. (**A**). Effects of different MFs on *E. coli* biofilm. (**B**). Effects of RMF on *E. coli* biofilm biomass under different exposure times. (**C**). Changes in cellular metabolic viability under different RMF exposure durations. (**D**). Changes in planktonic cell counts under different RMF exposure durations. *, *p* < 0.05. **, *p* < 0.01. ***, *p* < 0.001. Data were all expressed as mean ± SD (*n* = 6 for (**A**,**B**); *n* = 3 for (**C**,**D**)).

Previous studies have reported that 50–500 mT SMF reduced *E. coli* adhesion [22,45]. In this report, the effects of different MFs on *E. coli* biofilm were examined in a 96-well plate. The MFs used were PMF with 2.5 T and 15 pulses, RMF with 15 mT and 50 Hz, and SMF with 100 mT. The treatment time was 1 h for all the MFs. The results showed that PMF decreased biofilm biomass to 79.5% (±6.3%) relative to the control group, RMF to 75.3% (±13.2%), and SMF to 85.1% (±8.8%) (Figure 2A). These results indicated that MFs with different intensities and types reduced the biofilm of *E. coli*.

Furthermore, different treatment durations were tested under RMF to estimate its effect. Nine plates were placed in the processing chamber for 8 h, and eight of them were placed in RMF for different durations. The results showed that biofilm biomass was significantly reduced to 75% (±7.1%) of the control at 1 h and further decreased to 68.3% (±6.3%) at 2 h (Figure 2B). It partially recovered to 78.7% (±10.9%) at 3 h and stayed at 76.3–82.5% from 4 to 8 h, still comparable to the 3 h group (Figure 2B). Metabolic activity assays and planktonic cell counting were also performed. Metabolic activity only dropped significantly to 83.8% (±4.9%) of control at 2 h, while other time points showed no significant difference versus control (Figure 2C). Planktonic cell counts increased significantly at 1 h and showed non-significant decreases at 2 h and 3 h (Figure 2D). Other exposure times showed no significant difference from the control (Figure 2D). These results demonstrate that 1 h RMF reduces *E. coli* biofilm biomass without obvious loss of metabolic activity. To further explore the direct mechanism underlying this phenomenon, the following experiments were performed under 1 h RMF exposure, unless otherwise specified.

### 3.2. Observation of MF Effects on E. coli Biofilm on Glass Slides

To directly observe *E. coli* biofilm, glass slides were inserted into the bacterial solution, cultured for 48 h, and treated by MF. Consistent with previous results, *E. coli* cells adhered to glass slides and formed biofilms in control groups, while the adherent cells were significantly reduced in all treatment groups (Supplementary Figure S2).

**Figure 3:**
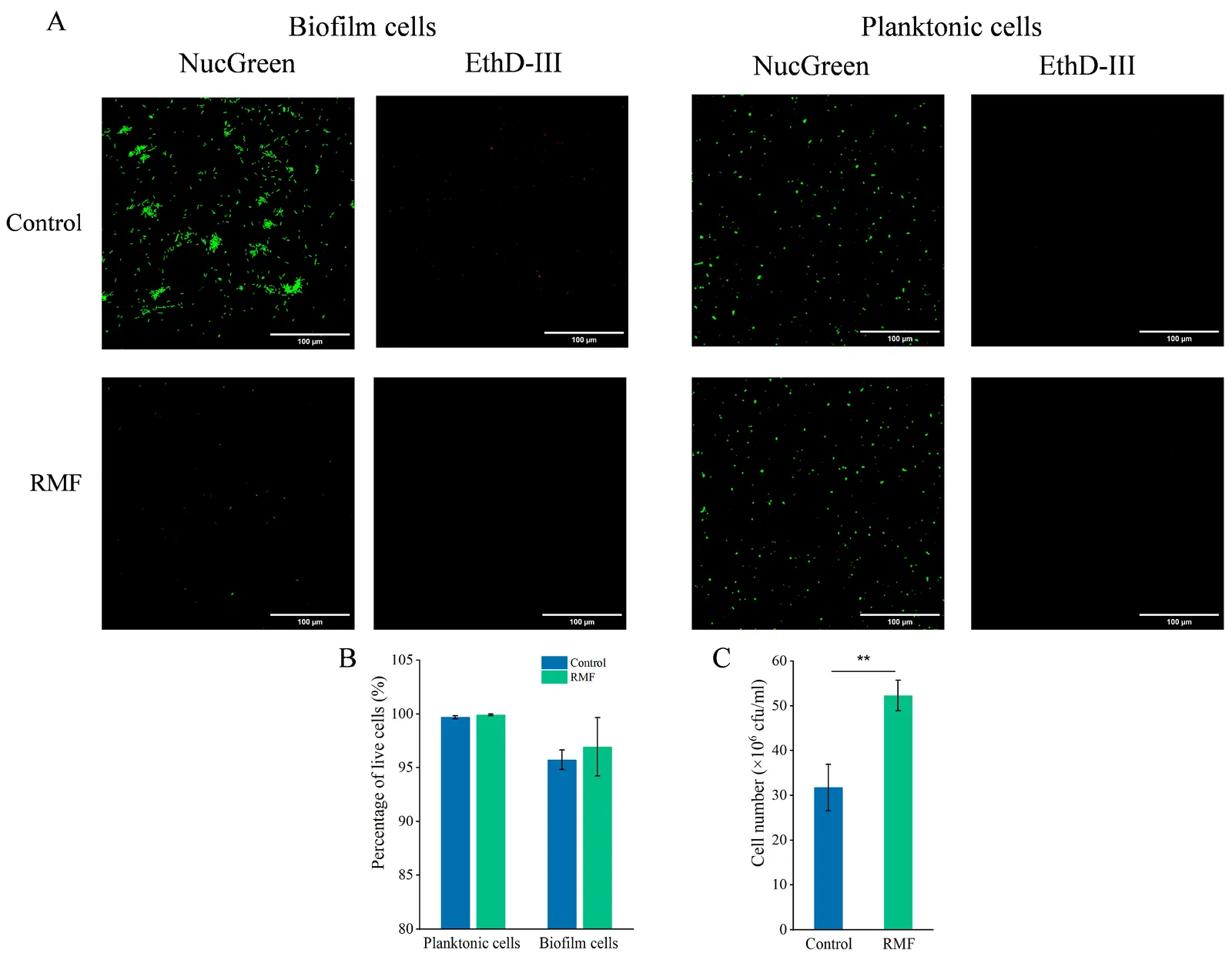
Observation and plate counting of live *E. coli* cells. (**A**). Live/dead cell staining images of biofilm and planktonic cells after 1 h of RMF treatment. NucGreen (membrane-permeable) stains live and dead cells. EthD-III (membrane-impermeable) only labels cells with compromised membranes. The staining incubation time was 15 min. The scale bar is 100 μm. These images are representative images of multiple samples. (**B**). Quantification of fluorescence images. (**C**). Plate counting of planktonic cells. **, *p* < 0.01. Data are expressed as mean ± SD (*n* = 3 for (**B**,**C**)).

Cell viability of *E. coli* biofilm and planktonic cells after 1 h RMF treatment was evaluated via live/dead cell staining. This assay is based on the detection of bacterial membrane permeabilization. Fluorescent signals from NucGreen revealed abundant adherent cells on glass slides in the untreated control group, whereas only a few adherent cells were observed in the RMF-treated group (Figure 3A). Numerous planktonic cells were observed in both the untreated control and RMF-treated groups (Figure 3A). Notably, the fractions of cells with compromised membranes were consistently low across all groups and showed no correlation with RMF exposure (Figure 3A,B), meaning that RMF treatment did not cause cell death. Plate counting showed that the number of planktonic cells increased significantly (Figure 3C). These results indicated that RMF treatment reduced *E. coli* biofilm by transforming cells from the biofilm state to the planktonic state.

### 3.3. Expression Changes in E. coli Biofilm-Related Genes Under MF Treatment

This transformation from biofilm state to planktonic state was accompanied by gene expression changes. In our previous work [46], transcriptome sequencing was performed and described systematically to explore DEGs between the PMF treatment group and the untreated group. Transcriptome analysis revealed 1128 DEGs, with 920 DEGs downregulated and 208 DEGs upregulated (Supplementary Materials S3). In this study, further analysis revealed that multiple genes directly related to *E. coli* biofilm formation, such as *pgaABCD*, were downregulated with PMF treatment (Table 1). Additionally, some related regulatory genes, such as *csrA*, were upregulated (Table 1). Further analysis of the transcriptome showed that the periplasmic stress-related transcription factor RpoE was activated under magnetic field treatment, with upregulated expression of multiple downstream genes (Table 1). The expression levels of these upregulated genes changed within the range of 2–3-fold. Both CsrA and RpoE inhibit *E. coli* biofilm formation [37,39], and MF may inhibit *E. coli* biofilm formation through these two pathways.

**Figure 4:**
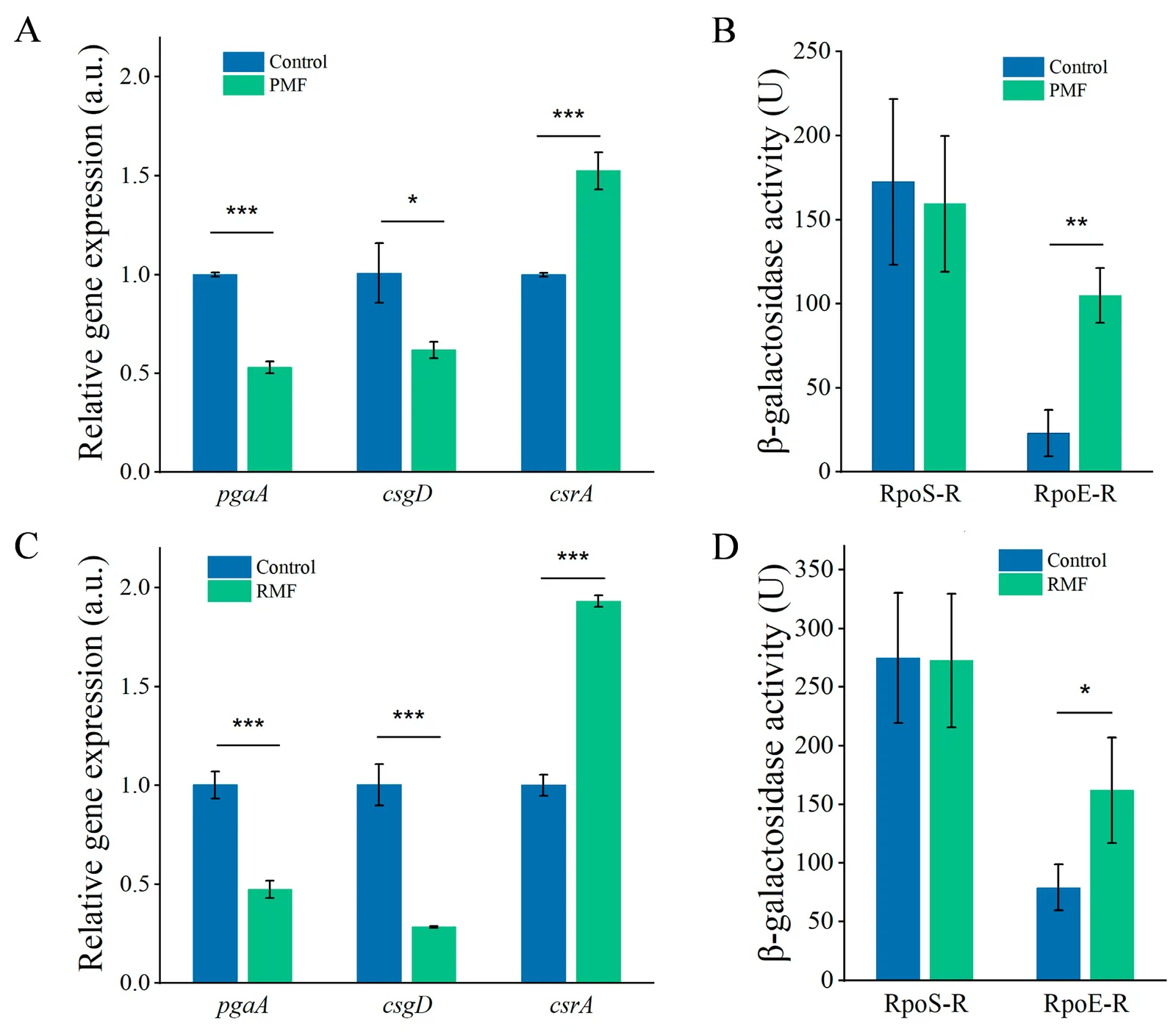
The relative expression of biofilm-related genes and promoter activity regulated by RpoS and RpoE with or without treatment with PMF or RMF. (**A**). The relative expression of biofilm-related genes with or without PMF treatment. (**B**). The changes in promoter activity regulated by RpoS and RpoE with or without PMF treatment. (**C**). The relative expression of biofilm-related genes with or without PMF treatment. (**D**). The changes in promoter activity regulated by RpoS and RpoE with or without PMF treatment. RpoS-R, the promoter activity regulated by RpoS; RpoE-R, the promoter activity regulated by RpoE. *, *p* < 0.05. **, *p* < 0.01. ***, *p* < 0.001. Data are all expressed as mean ± SD (*n* = 3 independent replicates).

Because transcriptome sequencing was performed on shake-flask-cultured planktonic cells, qRT-PCR was used to validate expression changes in *pgaA*, *csgD,* and *csrA* in biofilm-forming cells with or without PMF and RMF treatment. The *rpoD* gene was used as a housekeeping gene. In the PMF treatment group, the mRNA abundance of *pgaA* was reduced to 0.530 (±0.030)-fold relative to the control, *csgD* mRNA abundance reached 0.618 (±0.042)-fold of the control, and *csrA* mRNA abundance increased to 1.525 (±0.095)-fold of the control (Figure 4A). In the RMF treatment group, the mRNA level of *pgaA* decreased to 0.475 (±0.043)-fold of the control, *csgD* mRNA to 0.283 (±0.005)-fold of the control, and *csrA* mRNA increased to 1.933 (±0.030)-fold of the control (Figure 4C). These results indicated that the effects of PMF and RMF on these genes were similar in shake flask-cultured cells and biofilm-forming cells.

The activity of the promoter regulated by RpoE was detected via β-galactosidase in biofilm-forming cells with or without PMF and RMF treatment. The activity of the promoter regulated by RpoS was also detected for comparison. RpoE-regulated promoter activity was elevated in both groups, reaching 4.584 (±0.719)-fold of the control for PMF (Figure 4B) and 2.049 (±0.568)-fold for RMF (Figure 4D). However, the RpoS-regulated promoters were continuously activated in both treatment and control groups, but they were not influenced by any MF (Figure 4B,D). These results indicated that PMF and RMF treatments triggered periplasmic stress in *E. coli* without inducing a general stress response.

In summary, PMF and RMF activate transcription factor RpoE and upregulate *csrA* expression, which may cause downregulation of biofilm-related genes and biofilm dispersion.

### 3.4. RMF Reduces E. coli Biofilms via Transcription Factor RpoE

The inhibition of biofilm by RpoE is achieved through *rybB*, which is a small regulatory RNA promoting the degradation of *csgD* mRNA [39]. But *rybB* is only 79 bp and cannot be detected by qRT-PCR. To further verify the role of transcription factor RpoE in the effect of MF on biofilm, strains overexpressing or knocking out related genes were constructed. The *rpoE* gene is essential for some *E. coli* strains [47] and could not be knocked out by the CRISPR/Cas9 technology used in this study. Instead, its negative regulatory genes *rseAB* were overexpressed. The degree of gene overexpression was qualitatively assessed by SDS-PAGE (Supplementary Figure S3A). The biofilms of these strains were treated with RMF and evaluated.

**Figure 5:**
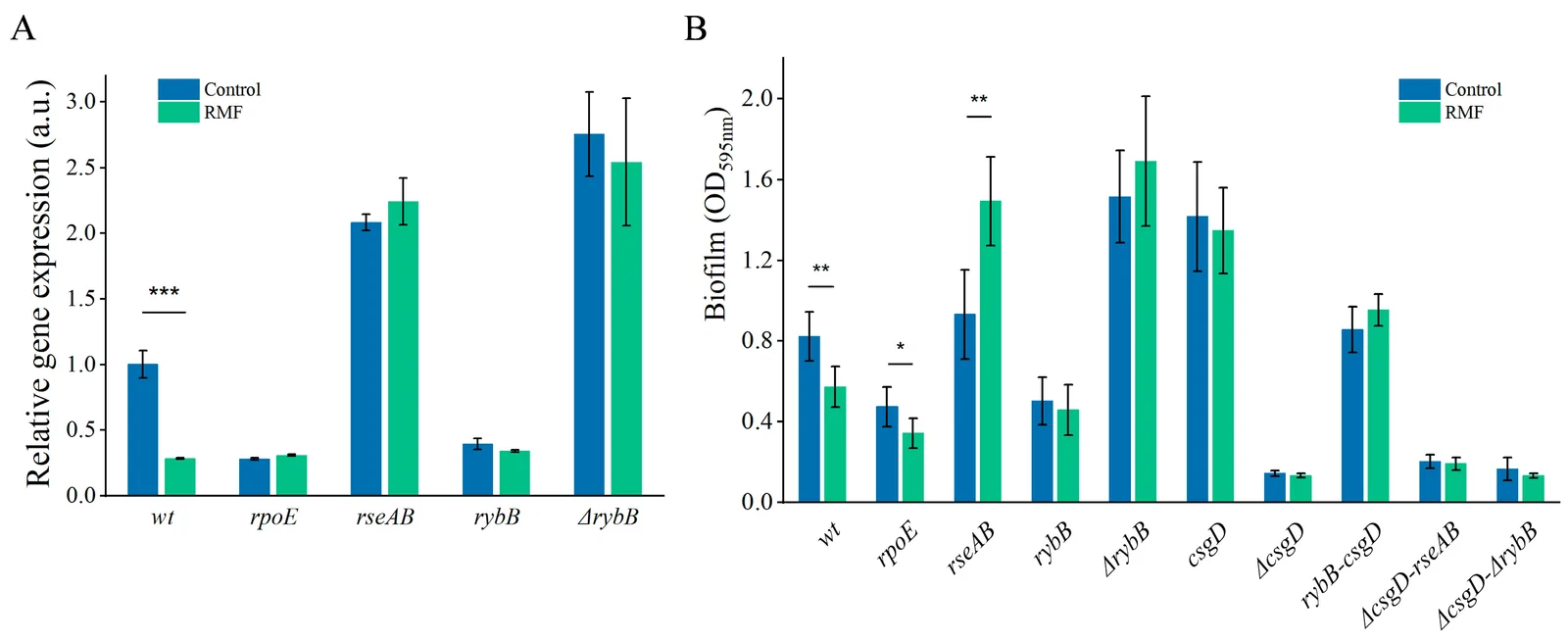
The effects of overexpression and knockout of *rpoE-related* genes on RMF-induced biofilm reduction. (**A**). The relative expression of *csgD* in different strains with or without RMF treatment. (**B**). Quantitative analysis of crystal violet staining of the biofilm formed by *rpoE*-related gene mutant strains with or without RMF treatment. *, *p* < 0.05. **, *p* < 0.01. ***, *p* < 0.001. Data are all expressed as mean ± SD (*n* = 3 for (**A**) and *n* = 6 for (**B**)).

The *csgD* expression levels of some strains were detected (Figure 5A). Overexpression of *rpoE* and *rybB* both suppressed the expression of *csgD*, reducing it to 0.280 (±0.009)-fold and 0.395 (±0.041)-fold of the control (Figure 5A), respectively. In contrast, overexpression of *rseAB* and knockout of *rybB* promoted *csgD* expression, increasing it to 2.084 (±0.061)-fold and 2.755 (±0.329)-fold of the control (Figure 5A), respectively. The *csgD* expression of all these strains was not altered by RMF treatment, in contrast to the wild-type strain (Figure 5A), suggesting that *rpoE* and *rybB* participate in the RMF-induced downregulation of *csgD*.

Furthermore, both *rpoE* and *rybB* overexpression inhibited biofilm formation (Figure 5B). RMF treatment induced a further biofilm reduction in these overexpression strains, especially in the *rpoE*-overexpression strain (Figure 5B), indicating that other regulatory pathways such as the *csrA* pathway remain functional. On the contrary, *rseAB* overexpression and *rybB* knockout significantly increased biofilm formation (Figure 5B). The biofilm of these strains further increased with RMF treatment, especially in the *rseAB* overexpression strain (Figure 5B), indicating that other effects of RMF promoted biofilm formation. *csgD* overexpression promoted biofilm formation and rescued the biofilm reduction caused by *rybB* overexpression (Figure 5B). However, the strain with simultaneous overexpression of both *csgD* and *rpoE* could not grow in liquid medium. *csgD* knockout strongly inhibited biofilm formation, which could not be rescued by *rseAB* overexpression and *rybB* knockout (Figure 5B). In addition, the biofilms of all strains with altered *csgD* expression were not affected by RMF (Figure 5B). The detailed changes in biofilm-forming capacity of genetically modified strains and RMF effects on these strains are shown in Table 2. These results indicated that *rpoE*, *rybB,* and *csgD* affected *E. coli* biofilm formation and were responsible for RMF-induced biofilm reduction.

### 3.5. RMF Reduces E. coli Biofilm via Metabolic Regulator CsrA

CsrA inhibits biofilm formation by binding to *pgaA* mRNA and preventing translation [37], which makes the mRNA unstable [48]. Since *rpoE* overexpression could not completely eliminate RMF-induced biofilm reduction, *csrA* may also play a role in this process. To verify this, *csrA* and related genes were overexpressed or knocked out. The *csrA* gene is also essential for some *E. coli* strains [47] and could not be knocked out by the CRISPR/Cas9 technology used in this study. *csrB* is the inhibitory gene of *csrA* [40] and was overexpressed. The degree of gene overexpression was qualitatively assessed by SDS-PAGE or qRT-PCR (Supplementary Figure S3). The biofilms of these strains were treated with RMF and evaluated.

**Figure 6:**
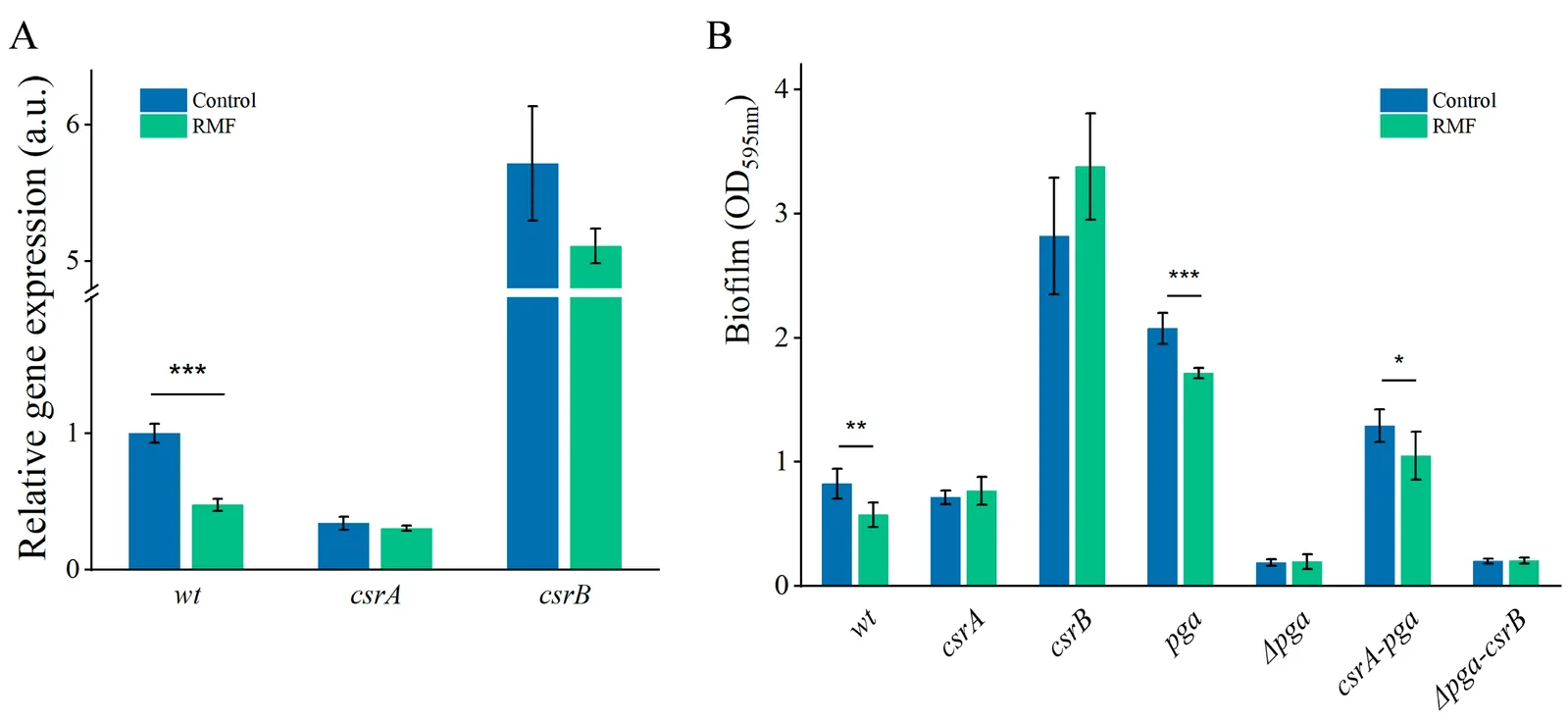
The effects of overexpression and knockout of *csrA*-related genes on RMF-induced biofilm reduction. (**A**). The relative expression of *pgaA* in different strains with or without RMF treatment. (**B**). Quantitative analysis of crystal violet staining of the biofilm formed by *csrA*-related gene mutant strains with or without RMF treatment. *, *p* < 0.05.**, *p* < 0.01. ***, *p* < 0.001. Data are all expressed as mean ± SD (*n* = 3 for (**A**), and *n* = 6 for (**B**)).

The *pgaA* expression levels of *csrA*- and *csrB*-overexpressing strains were detected. Overexpression of *csrA* downregulated *pgaA* mRNA levels, reducing them to 0.341 (±0.047)-fold of the control (Figure 6A). In contrast, overexpression of *csrB* elevated *pgaA* mRNA levels, increasing them to 5.717 (±0.420)-fold of the control (Figure 6A). Moreover, *pgaA* expression in both strains did not change under RMF treatment (Figure 6A), indicating the function of *csrA* in the PMF-induced *pgaA* downregulation.

Overexpression of *csrA* did not significantly reduce biofilm formation (Figure 6B), possibly owing to tight post-transcriptional control of CsrA activity [40]. However, its overexpression abolished RMF-induced biofilm reduction, suggesting an important regulatory role in this process. Overexpression of *csrB* significantly increased biofilm formation (Figure 6B), and this improvement was far greater than *rseAB* overexpression and Δ*rybB*. This result showed that CsrA was more important in biofilm regulation than RpoE, whose main function is the repair of misfolded periplasmic proteins. Meanwhile, the *csrB* overexpression strain showed increased biofilm with RMF treatment (Figure 6B), similar to *rseAB*-overexpression and Δ*rybB* strains. Furthermore, *pgaA* overexpression promoted biofilm formation, even in co-expression with *csrA* (Figure 6B). However, the biofilms of both strains were significantly reduced with RMF treatment, which might be caused by CsrA protein activation, as supported by *csrB* downregulation (Supplementary Figure S4). *pgaA* knockout strongly inhibited biofilm formation, which could not be rescued by *csrB* overexpression (Figure 6B). The detailed changes in biofilm-forming capacity of genetically modified strains and RMF effects on these strains are also shown in Table 2.

These results indicated that both the CsrA pathway and RpoE pathway were necessary for RMF-induced biofilm inhibition. Because these two pathways inhibit *E. coli* biofilm in different ways, disruption of either pathway abolished the inhibitory effect. However, current results cannot distinguish whether these two pathways function independently or exhibit cross-talk.

### 3.6. RMF Affects Different Bacterial Biofilms

**Figure 7:**
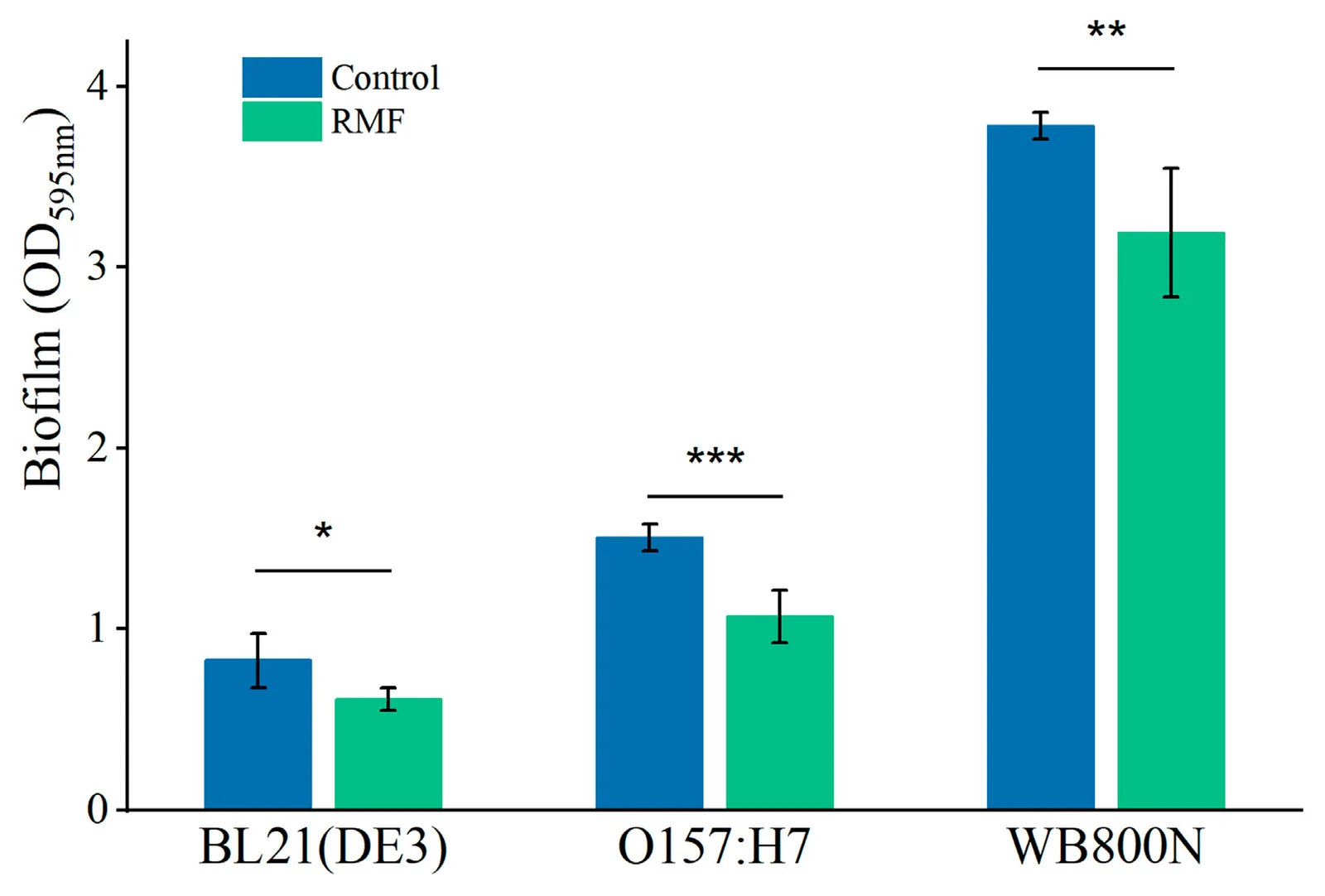
The effects of RMF on different bacterial biofilms. Quantitative analysis was conducted by crystal violet staining. *, *p* < 0.05. **, *p* < 0.01. ***, *p* < 0.001. Data are all expressed as mean ± SD (n = five independent replicates).

The previous experiments were conducted using *E. coli* BL21(DE3), a commonly used and non-pathogenic laboratory strain. Whether RMF exerts similar antibiofilm effects in other *E. coli* strains or bacterial species therefore remains to be determined. To evaluate the broader applicability of RMF treatment, the effects of 1 h RMF exposure on biofilm formation by *E. coli* O157:H7 and *Bacillus subtilis* WB800N were examined. RMF treatment reduced the biofilm biomass of *E. coli* O157:H7 to 70.9% (±9.7%) of that of the control and that of *B. subtilis* WB800N to 84.3% (±9.4%) (Figure 7). These results indicate that the inhibitory effect of RMF on biofilm formation is not restricted to *E. coli* BL21(DE3). However, mechanistic analyses were not performed in these additional strains. Given the conserved and important regulatory roles of CsrA and RpoE in *E. coli* [49,50], the pathways identified in *E. coli* BL21(DE3) may also contribute to the RMF-induced reduction in biofilm formation in *E. coli* O157:H7. In contrast, *B. subtilis* employs distinct regulatory networks for biofilm development, and the mechanisms underlying its response to RMF require further investigation.

## 4. Discussion

**Figure 8:**
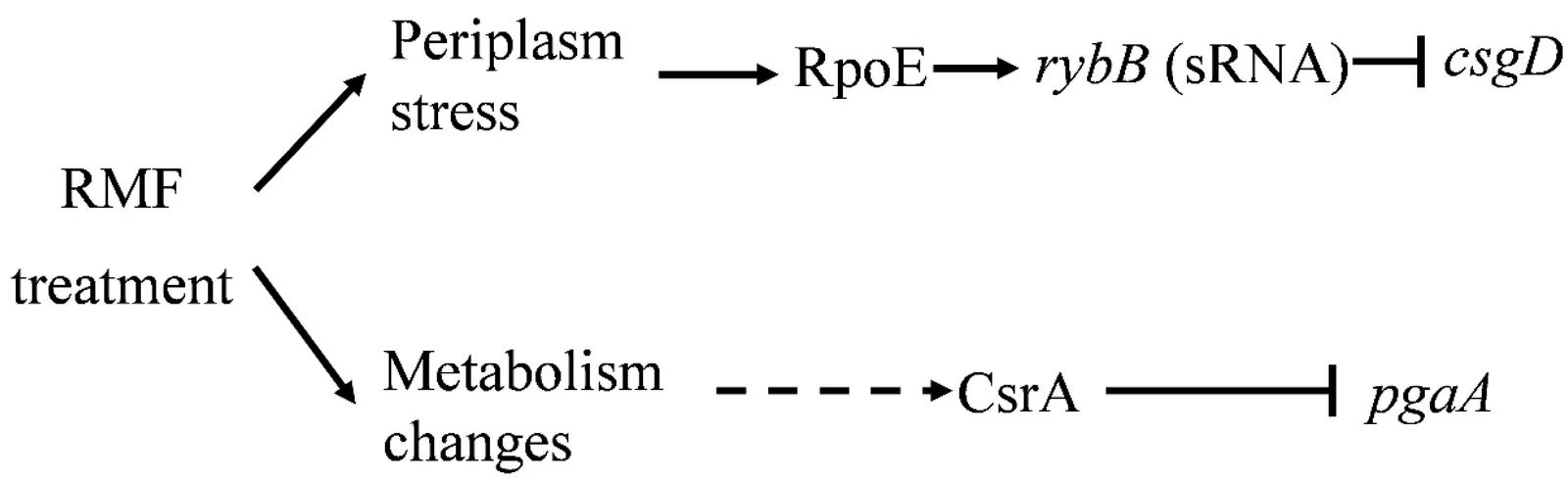
Schematic of the regulatory pathway of RMF effects on *E. coli* biofilm. The dashed line represents speculation.

In this study, we found that 1 h RMF treatment could disperse *E. coli* biofilm and increase planktonic cells without obvious viability loss. Further exploration demonstrated that this occurred via the downregulation of *csgD* and *pgaA*, which was caused by the periplasmic stress transcription factor RpoE and the carbon metabolism regulator CsrA, respectively (Figure 8). Inhibition of either regulatory pathway or knockout of *csgD* or *pgaA* abolished the biofilm inhibitory effect of RMF. In addition, biofilm reduction also occurred in various MF treatments and species. Their mechanisms need further exploration, and this study can provide some reference.

The effects of MFs on organisms have been studied for a long time [51,52]. Many studies have demonstrated that MFs with different intensities affect bacterial metabolism [52,53], including *E. coli* metabolism [46,54]. CsrA is a regulator of carbohydrate metabolism. However, the transcriptional regulation of *csrA* remains poorly characterized [55], and its direct transcriptional activator has not yet been identified. The upregulation of *csrA* under MFs may be caused by metabolic changes, which is speculated based on its function. The effect of MFs on the periplasmic stress transcription factor RpoE has been proposed for the first time. RpoE is activated by increased expression and misfolding of outer membrane proteins [56]. Its inducers include heat shock, hyperosmotic shock, metal ion exposure, and changes in the outer membrane lipopolysaccharide structure. Some studies show that MFs affect the composition of bacterial cell membranes [57,58] and cell membrane potential [59], which may be related to the activation of RpoE. Thus, MF-dependent activation of these two pathways is biologically plausible, yet the underlying molecular processes require further experimental investigation. In addition, single mutant assays in this study cannot determine whether the two pathways are independent or have cross-talk. Future experiments constructing double-mutant strains, alongside assays to test how mutation of one gene affects expression of the other, will help dissect the epistatic relationship between the RpoE-RybB and CsrA pathways.

Many previous studies have shown that MF damage suppresses microbial growth via oxidative stress induction [26] and cell membrane impairment [27]. After 2 h RMF exposure, we also detected simultaneous decreases in *E. coli* biofilm biomass, metabolic activity, and planktonic cell numbers (Figure 2), suggesting cellular damage at this time point. This injury recovered during longer treatment (Figure 2), revealing a “window effect” for RMF. Importantly, the biofilm biomass reduction, the primary endpoint of this study, was already detectable at 1 h RMF treatment without a decrease in metabolic activity or planktonic cell counts. This study has proposed an explanation for this phenomenon. The later cellular damage and adaptive responses induced by RMF may involve separate mechanisms and require further investigation.

In biofilm tests with genetically engineered strains, strains with repressed RpoE and CsrA pathways (the *rseAB* overexpression, Δ*rybB* knockout, and *csrB* overexpression) displayed elevated biofilm biomass after 1 h RMF treatment. This suggests RMF also acts on additional biofilm regulatory circuits that promote biofilm formation, such as the second messenger c-di-GMP and general stress regulator RpoS. c-di-GMP accumulates under various environmental stresses and promotes biofilm formation [60], yet whether RMF alters c-di-GMP levels remains unknown. RMF may induce c-di-GMP accumulation, but this biofilm-promoting effect may be normally concealed by the inhibition of RpoE and CsrA. RpoS is a global stress-responsive sigma factor. While RpoS activity did not change significantly under 1 h RMF in wild-type cells, it may become activated in strains deficient in RpoE or the CsrA pathway to enhance biofilm production. RpoS may also participate in the cellular injury and adaptive recovery observed during longer RMF exposure. Future quantification of intracellular c-di-GMP and RpoS activity in different mutant strains over a time course of RMF treatment will clarify these mechanisms.

In addition, biofilm development and maintenance rely on the coordinated interplay between biofilm-attached cells and planktonic subpopulations. However, the qRT-PCR data in this work reflect the collective response of the whole biofilm community rather than subpopulation-specific transcriptional profiles. Future work will isolate biofilm-associated and planktonic fractions to resolve their distinct responses to RMF treatment.

This study can provide some reference for the mechanism of action of MFs on other bacterial biofilms. It is reasonable to infer that MFs of varying intensities and types may trigger comparable early cellular responses, including periplasmic stress and metabolic reprogramming, which could modulate biofilm formation in other bacterial species. For instance, it can be speculated that the biofilm reduction in *B. subtilis* by RMF treatment may be caused by the energy-related sigma factor SigB [61], which requires further verification. Further research is also needed to determine the effects of MFs on more pathogenic species, especially those clinical isolates with stronger biofilm-forming capacity [62].

In practical environments, microbial biofilms can develop at hard-to-treat locations, including the surface or interior of bulk-shipped food [4] and within infected patients [6], limiting the use of many biofilm removal approaches. MF can penetrate biological surfaces to reach inner regions and thus offer advantages for these complex environments. While high-intensity MF devices are large and hard to implement, low-intensity magnetic fields are easily obtained. However, naturally occurring biofilms feature more diverse bacterial species and structural architectures compared with laboratory monoculture biofilms [63]. Additional studies are needed to validate the effects of low-intensity MF on such real-world biofilms.

## Figures and Tables

**Table 1:** Changes in the expression of genes related to biofilm with PMF treatment.

Gene	Fold Change	Description
*pgaA*	0.374768	Partially deacetylated poly-β-1,6-N-acetyl-D-glucosamine export porin, required for the synthesis of the linear polymer of β-1,6-N-acetylglucosamine residues (PGA)
*pgaB*	0.309633	Poly-β-1,6-N-acetyl-D-glucosamine N-deacetylase and β-1,6 glycoside hydrolase, required for the partial de-N-acetylation and hydrolysis of poly-β-1,6-N-acetyl-D-glucosamine (known as PGA or PNAG)
*pgaC*	0.305207	Poly-*N*-acetyl-D-glucosamine synthase subunit PgaC, required for the synthesis of PGA
*csgB*	0.41873	Minor subunit of curlin, triggering the polymerization of CsgA to form curli fibers on the cell surface
*csgC*	0.380541	Curlin chaperone, maintaining the curlin subunit CsgA in a soluble monomeric state
*csgD*	0.298471	Curlin subunit gene D, which is a transcriptional regulator regulating a number of genes involved in the Curli assembly, transport, and structural components
*ycgR*	0.409126	Flagellar brake protein, leading to slower flagellar motor speeds by increasing c-di-GMP
*wza*	0.300973	Outer membrane polysaccharide export protein, involved in a CA export system
*luxS*	2.046398	S-ribosylhomocysteine lyase, involved in biosynthesis of autoinducer-2 (AI-2)
*csrA*	2.14606	Carbon storage regulator, affecting glycogen biosynthesis, glycogen degradation, gluconeogenesis, glycolysis, and biofilm formation
*ompR*	2.065953	Controlling the transcriptional expression of several genes involved in adaptation to changes in osmolarity and promoting biofilm formation
*rpoH*	2.030391	RNA polymerase sigma factor RpoH, controlling the heat shock response during log-phase growth and able to be transcribed from an RpoE-driven promoter
*rpoN*	2.042395	RNA polymerase sigma factor RpoN, controlling expression of nitrogen-related genes and able to be transcribed from an RpoE-driven promoter
*rseP*	2.003195	Intramembrane zinc metalloprotease RseP, catalyzing hydrolytic cleavage of peptide bonds within the hydrophobic lipid bilayer and required for activation of RpoE; only transcribed from an RpoE-driven promoter

**Table 2:** Changes in biofilm formation of different strains and their biofilm responses to 1 h RMF treatment. Percentage values for biofilm-forming capacity were calculated relative to the wild-type (wt) strain. Percentage values for biofilm responses to RMF treatment were calculated relative to the untreated group of the same strain. Corresponding *p*-values were calculated using the same comparison strategy. Data are presented as mean ± standard deviation (SD) from six independent replicates.

Strain	Biofilm-Forming Capacity			Biofilm Responses to RMF Treatment		
	Raw Value (Mean ± SD)	*p*-Value	Percentage (%) (Mean ± SD)	Raw Value (Mean ± SD)	*p*-Value	Percentage (%) (Mean ± SD)
wt	0.823 ± 0.121	1	100.04 ± 14.66	0.574 ± 0.100	0.0029	69.70 ± 12.13
*rpoE*	0.475 ± 0.098	0.0003	57.70 ± 11.96	0.344 ± 0.074	0.0259	72.37 ± 15.49
*rseAB*	0.932 ± 0.221	0.3126	113.30 ± 26.81	1.493 ± 0.219	0.0013	160.20 ± 23.55
*csrA*	0.714 ± 0.055	0.0701	86.72 ± 6.63	0.768 ± 0.113	0.3175	107.50 ± 15.80
*rybB*	0.504 ± 0.118	0.0009	61.18 ± 14.36	0.459 ± 0.124	0.5372	91.04 ± 24.58
*csrB*	2.818 ± 0.470	0.0000	342.37 ± 57.14	3.377 ± 0.429	0.0567	119.85 ± 15.22
Δ*rybB*	1.515 ± 0.228	0.0001	184.06 ± 27.68	1.690 ± 0.320	0.3005	111.55 ± 21.14
*csgD*	1.417 ± 0.270	0.0006	172.12 ± 32.79	1.347 ± 0.213	0.6329	95.09 ± 15.02
Δ*csgD*	0.144 ± 0.014	0.0000	17.53 ± 1.67	0.133 ± 0.010	0.1554	92.70 ± 7.16
*rybB-csgD*	0.856 ± 0.114	0.6364	104.05 ± 13.86	0.955 ± 0.079	0.1122	111.54 ± 9.18
Δ*csgD-rseAB*	0.203 ± 0.033	0.0000	24.69 ± 3.96	0.192 ± 0.031	0.5726	94.79 ± 15.45
Δ*csgD-*Δ*rybB*	0.167 ± 0.057	0.0000	20.23 ± 6.88	0.133 ± 0.011	0.1813	79.45 ± 6.55
*pga*	2.076 ± 0.126	0.0000	252.25 ± 15.28	1.714 ± 0.042	0.0001	82.58 ± 2.03
Δ*pgaA*	0.190 ± 0.028	0.0000	23.11 ± 3.40	0.198 ± 0.060	0.7723	104.33 ± 31.50
*csrA-pga*	1.291 ± 0.131	0.0001	156.88 ± 15.87	1.051 ± 0.192	0.0297	81.40 ± 14.88
Δ*pgaA-csrB*	0.201 ± 0.020	0.0000	24.39 ± 2.40	0.206 ± 0.024	0.7103	102.28 ± 12.08

## Data Availability

The transcriptome data generated in this study for Table 1 have been deposited at Gene Expression Omnibus under accession codes GSE278595. The raw data supporting the conclusions of this article will be made available by the authors on request.

## Supplementary Materials

The following supporting information can be downloaded at: https://www.mdpi.com/article/10.3390/microorganisms14102200/s1, Supplementary Materials S1: Supplementary figures and supplementrary tables. Figure S1: The detailed spatial distribution of RMF and SMF intensity. Figure S2: Crystal violet staining and quantification of *Escherichia coli* biofilms formed on glass slides. Figure S3: SDS-PAGE and qRT-PCR results for gene overexpression. Figure S4: The effects of PMF and RMF treatment on csrB expression. Data were all expressed as mean ± SD (n = 3). Table S1: Bacterial strains and plasmids used in this study. Table S2: The primers used in this study. Table S3: Genomic coordinates of the genes used in this study. Table S4: Links to the software used in this study. Supplementary Materials S2: Gene sequencing information. Supplementary Materials S3: Lists of differentially expressed genes identified by transcriptome analysis, with the results of GO enrichment and KEGG enrichment analyses. Supplementary Methods: Detailed procedures for strain construction, including gene overexpression and gene knockout.

## Abbreviations

The following abbreviations are used in this manuscript:

MF	Magnetic field
PMF	Pulse magnetic field
RMF	Rotating magnetic field
SMF	Static magnetic field
DEGs	Differentially expressed genes
