# Supplementary material for: Magnetic Fields Disperse Escherichia coli from Biofilm via CsrA and RpoE Regulation
Source: Microorganisms. 2026 Oct 1;14(10):2200. doi: 10.3390/microorganisms14102200 (PMC13707543; doi:10.3390/microorganisms14102200)
Supplement: Supplementary file 1 [file microorganisms-14-02200-s001.zip › Supplementary Materials S1.pdf]

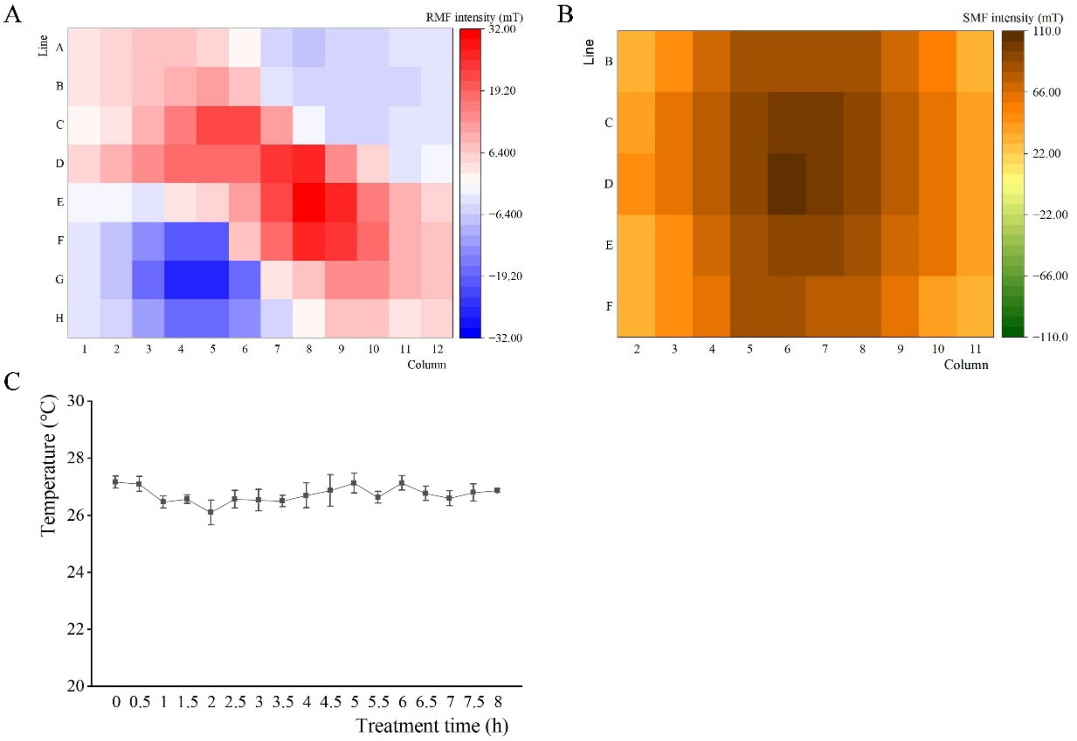

Supplementary Figure S1. The detailed spatial distribution of RMF and SMF intensity. A Spatial distribution of MF intensity across the 96-well plate placement area at a single time point under RMF treatment. Positive values indicate MF lines pointing outward, while negative values indicate MF lines pointing inward. The region used in the experiments spans wells B2 to G11. The maximum magnetic field intensity within this region is approximately 31.52 mT, and the minimum intensity is about 0.72 mT. The MF rotates at a frequency of 50 Hz during operation with no obvious vibration. B Spatial distribution of MF intensity for the 96-well plate positioned above the magnet under SMF treatment. All MF lines point outward. The experimental region used spans wells D5 to E7. Within this region, the maximum magnetic field intensity is approximately 103.74 mT, and the minimum intensity is about 92.37 mT. C Temperature profile of the working area during the 8 h RMF treatment. n=3 for C.

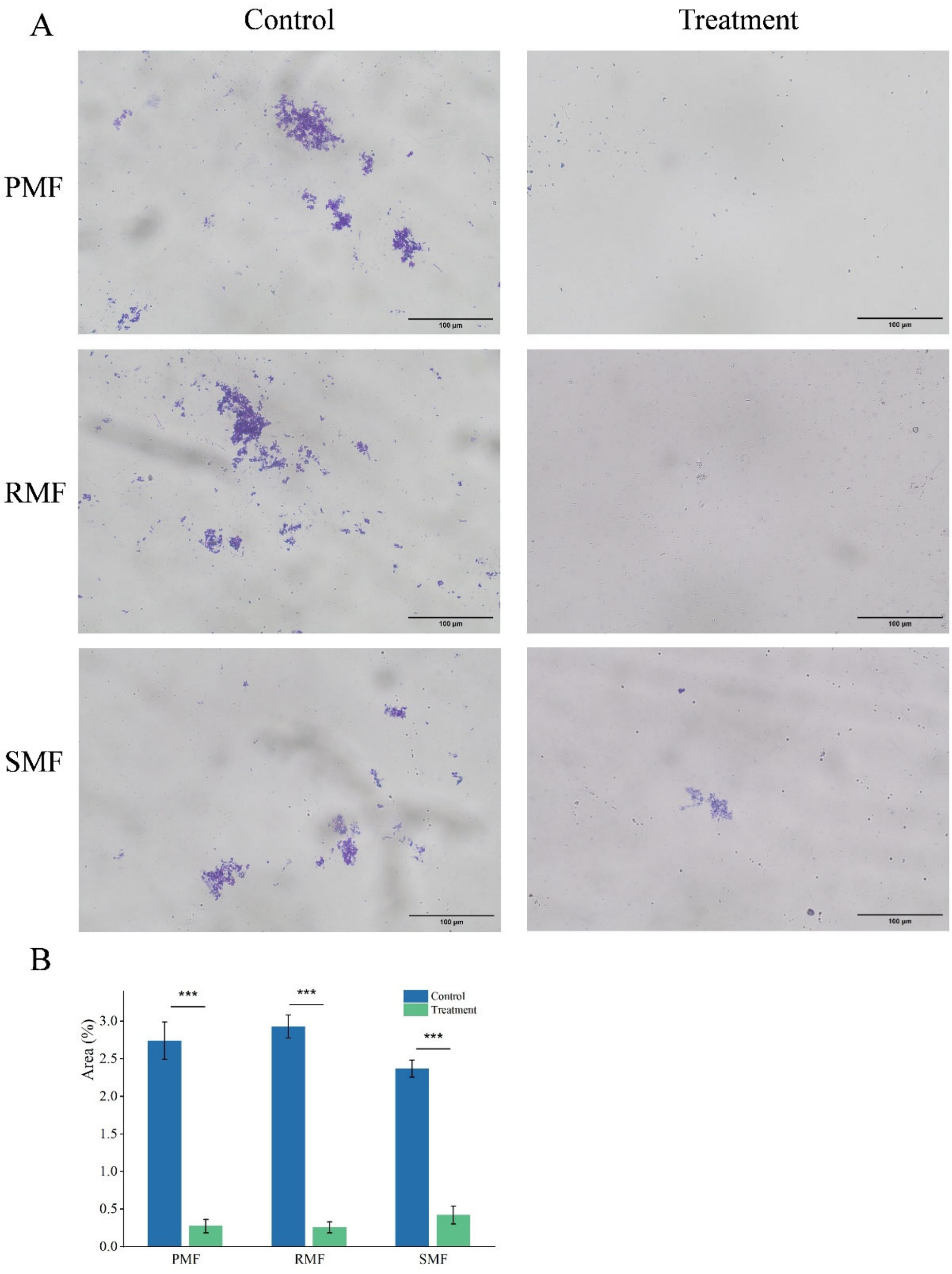

Supplementary Figure S2. Crystal violet staining and quantification of *Escherichia coli* biofilms formed on glass slides. A Microscopic image of the MF effects on *E. coli* biofilm. The scale bar is 100 μm. These images are representative images of multiple samples. B Quantification of crystal violet-stained images. ImageJ was used to calculate the stained area, which was normalized to the total area of each image. ***: $p < 0.001$. n=3 for B.

A

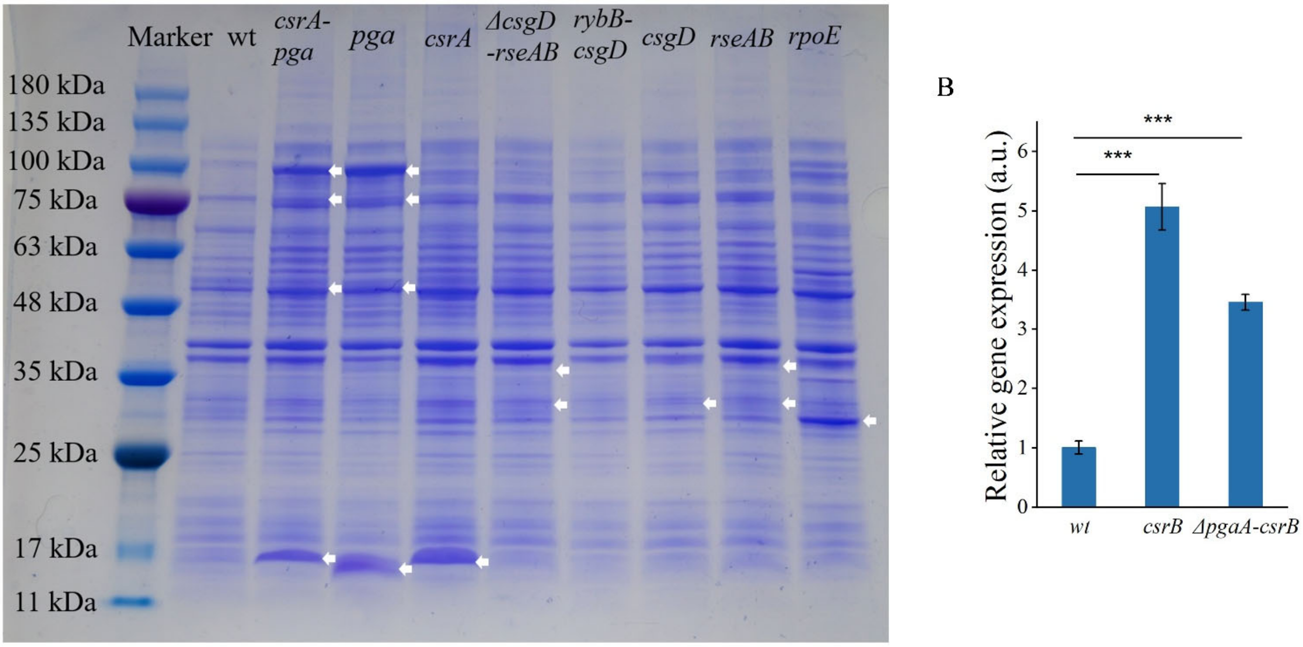

Supplementary Figure S3. SDS-PAGE and qRT-PCR results for gene overexpression. A. SDS-PAGE of gene overexpression strains. Each lane is labeled with the strain name, and the molecular weights of bands in the marker lane are indicated. White arrows mark the positions of overexpressed protein bands. The theoretical molecular masses of proteins fused with the intrinsic peptide encoded by the plasmid are as follows: RpoE, 26.1 kDa; RseA, 27.8 kDa; RseB, 35.7 kDa; CsgD, 28.4 kDa; CsrA, 10.4 kDa; PgaA, 95.6 kDa; PgaB, 77.4 kDa; PgaC, 50.7 kDa; PgaD, 16.1 kDa. The expression levels of RseA, RseB and CsgD were relatively low, and their bands were only slightly stronger than those of the control group. CsrA itself has a small molecular weight and cannot be visualized in this gel, whereas its dimeric protein band was clearly detectable. Other proteins exhibited bands markedly stronger than the control. B. Relative abundance of *csrB* RNA. The *csrB* strain showed a 5.1-fold increase compared with the control, and the *csrB*-Δ*pga* strain showed a 3.5-fold increase compared with the control. ***, $p<0.001$. n=3 for B.

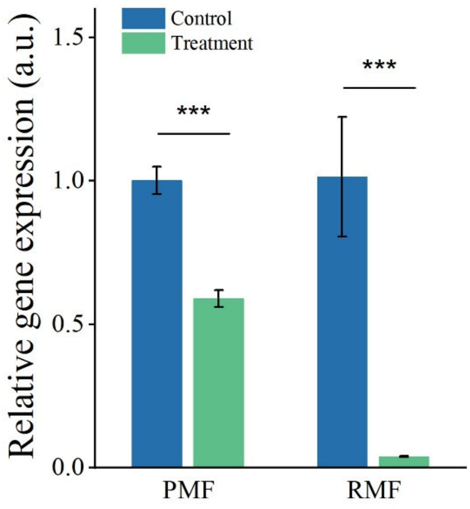

Fig S4. The effects of PMF and RMF treatment on *csrB* expression. Data were all expressed as mean ± SD (n = 3).

Supplementary Table S1. Bacterial strains and plasmids used in this study.

Strain/Plasmid	Genotype	Source
**Bacterial strains**		
wt	*E. coli* BL21(DE3)	Shanghai Weidi Biotechnology
DH5α	*E. coli* DH5α	Shanghai Weidi Biotechnology
O157:H7	*E. coli* O157:H7	Laboratory storage
WB800	*Bacillus subtilis* WB800N	Laboratory storage
rpoE	wt P_{T7}: *rpoE*	This study
rseAB	wt P_{T7}: *rseAB*	This study
csrA	wt P_{T7}: *csrA*	This study
rybB	wt P_{rybB}: *rybB*	This study
csrB	wt P_{csrB}: *csrB*	This study
Δ*rybB*	wt Δ*rybB*	This study
csgD	wt P_{T7}:*csgD*	This study
Δ*csgD*	wt Δ*csgD*	This study
rybB-csgD	wt P_{rybB}: *rybB* P_{T7}: *csgD*	This study
Δ*csgD-rseAB*	wt Δ*csgD* P_{T7}: *rseAB*	This study
Δ*csgD*-Δ*rybB*	wt Δ*csgD* Δ*rybB*	This study
pga	wt P_{T7}: *pgaABCD*	This study
Δ*pgaA*	wt Δ*pgaA*	This study
csrA-pga	wt P_{T7}: *csrA* P_{T7}: *pgaABCD*	This study
Δ*pgaA-csrB*	wt Δ*pgaA* P_{csrB}: *csrB*	This study
Δ*lacZ*	wt Δ*lacZ*	This study
RpoS-R	wt Δ*lacZ* P_{sdoC}: *lacZ*	This study
RpoE-R	wt Δ*lacZ* P_{rpoHp3}: *lacZ*	This study
LacZ-R	Δ*lacZ* pET-*lacZ* (promoterless *lacZ*)	This study
**Plasmids**		
pET-28a	Expression vector, T7 promoter, Kan^R	Novagen
pCas9	Cas9 expression plasmid, Kan^R	Laboratory storage
pTargetF	CRISPR guide RNA plasmid, $Spec^R$	Laboratory storage
pET-*rpoE*	pET-28a P_{T7}: *rpoE*, Kan^R	This study
pET-*rseAB*	pET-28a P_{T7}: *rseAB*, Kan^R	This study
pET-*rybB*	pET-28a P_{T7}: *rybB*, Kan^R	This study
pET-*csgD*	pET-28a P_{T7}: *csgD*, Kan^R	This study
pET-*csrA*	pET-28a P_{T7}: *csrA*, Kan^R	This study
pET-*csrB*	pET-28a P_{T7}: *csrB*, Kan^R	This study
pET-*gfp*	pET-28a P_{T7}: *gfp*, Kan^R	Laboratory storage
pET-*pgaABCD*	pET-28a P_{T7}: *pgaABCD*, Kan^R	This study

pET-*rybB-csgD*	pET-28a P_{rybB}: *rybB* P_{T7}: *csgD*, Kan^R	This study
pET-*csrA-pga*	pET-28a P_{T7}: *csrA* P_{T7}: *pgaABCD*, Kan^R	This study
pTargetF-*rybB*	pTargetF carrying *rybB* guide RNA, $Spec^R$	This study
pTargetF-*csgD*	pTargetF carrying *csgD* guide RNA, $Spec^R$	This study
pTargetF-*pgaA*	pTargetF carrying *pgaA* guide RNA, $Spec^R$	This study
pTargetF-*lacZ*	pTargetF carrying *lacZ* guide RNA, $Spec^R$	This study
pET-P0-*lacZ*	pET-28a without promoter in the MCS, contains *lacZ*, Kan^R	This study
pET-P*sod*	pET-28a backbone; the T7 promoter was replaced by the *sod* promoter, Kan^R	This study
pET-P*rpoH*p3-*lacZ*	pET-28a backbone; the T7 promoter was replaced by the *rpoHp3* promoter, Kan^R	This study
pET- P*sod* -*lacZ*	pET-P*sod* carring *lac*Z reporter gene, Kan^R	This study
pET-P*rpoH*p3-*lacZ*	pET-P*rpoH*p3 carring *lacZ* reporter gene, Kan^R	This study

Supplementary Table S2. The primers used in this study.

Primer	Sequence	Usage	Product Size	Restriction enzyme
rpoE1	CGGGATCCATGAGCGAGCAGTTAACGGA	*rpoE* cloning	602 bp	BamHI
rpoE2	AAGGAAAAAAGCGGCCGCTCAACGCCTGATAAGCGGT			NotI
rseAB1	CGGGATCCATGCAGAAAGAACAACTTTC	*rseAB* cloning	1624 bp	BamHI
rseAB2	CCCAAGCTTTCATTGCGCTGCCCCGAACT			HindIII
csrA1	CGGGATCCATGCTGATTCTGACTCGTC	*csrA* cloning	203 bp	BamHI
csrA2	CCCAAGCTTTTAGTAACTGGACTGCTGGG			HindIII
csrB1	GAAGATCTGATCTCTTACAGACCCTGTA	*csrB* cloning	467 bp	BglII
csrB2	CCCAAGCTTCGCAGTAACGCTTCAAGCGTC			HindIII
rybB1	GAAGATCTAACTGAAGTTGCCCTGAAAAT	*rybB* cloning	235 bp	BglII
rybB2	CCCAAGCTTGCCATCGTTAAAAAACAGTGC			HindIII
pga1	CGGGATCCATGTATTCAAGTAGCAGAA	*pgaA* cloning	6193 bp	BamHI
pga2	CCCAAGCTTTTATGCCCGGACTAGCGCTT			HindIII
csgD1	CGGGATCCATGTTTAATGAAGTCCATAGT	*csgD* cloning	668 bp	BamHI
csgD2	CCCAAGCTTTTATCGCCTGAGGTTATCGTT			HindIII
rpoHp1	GAAGATCTTGAACTTGTGGATAAAATCACGGTCTGATAAAACAGTGA ATGATAACCTCGTTG	Promoter rpoHp3 cloning	84 bp	BglII
rpoHp2	TGTTATCCGCTCACAATTCCGACAACGAGGTTATCATTCA			N/A
sodp1	GAAGATCTACGCCACTTTACGCCTGAATAATAAAAGC	Promoter sodp cloning	79 bp	BglII
sodp2	TGTTATCCGCTCACAATTCCGAGCAGGGAAAGAGTATAACACGCTTT TATTATTCA			N/A
lacZ1	GCGGATCCATGACCATGATTACGGATT	*lacZ* cloning	3095 bp	BamHI

lacZ2	ATAAGAATGCGGCCGCTTATTTTTGACACCAGACCA			HindIII
lacZ0	GAAGATCTATGACCATGATTACGGATT	*lacZ* cloning for pet-lacZ	3095 bp	BglII
Peo1	TGTGAGCGGATAACAATTCCTAGAAATAATTTTGTTTAA	Fusion fragment for promoter cloning	163 bp	N/A
Peo2	CGGAATTCGGATCCGCGACCCA			EcoRI
gfp1	ATGGTGAGCAAGGGCGAGGA	*gfp* cloning for *rybB* knockout	717 bp	N/A
gfp2	CTTGTACAGCTCGTCCAT			N/A
Sclone1	CCGGCGTAGAGGATCGAGATATAGGCGCCAGCAACCGCAC	Amplification of co-expression cassette	NA	N/A
Sclone2	TAATTTCGCGGGATCGAATCCGGATATAGTTCCTCCTT			N/A
ryF1	CAGTCTGGTGCCCGTGGTTAT	Upstream fragment for *rybB* knockout	317 bp	N/A
ryF2	TCCTCGCCCTTGCTCACCATACTAATTAAGACTGATGC			N/A
ryR1	ATGGACGAGCTGTACAAGAGGTTGATGGGTTTTTTGTT	Downstream fragment for *rybB* knockout	418 bp	N/A
ryR2	CGAAATGGAATGATTCTTCGC			N/A
Sgry1	AACCTTGAACCGAAATGGCGGTTTTAGAGCTAGAAATAGC	sgRNA for *rybB* knockout	2117 bp	N/A
Sgry2	AAACCGCCATTTCGGTTCAAGGTTACTAGTATTATACCTAGGAC			N/A
pgF1	ATTTCAGCCTGAGGAAGATCA	Upstream fragment for *pgaA* knockout	480 bp	N/A
pgF2	ATTAGAAATTCTGTGGCACGA			N/A
pgR1	TGCCACAGAATTTCTAATCCTGTATTACTCCATGTATT	Downstream fragment for *pgaA* knockout	489 bp	N/A
pgR2	GATGATGATAGTTGCTGAAT			N/A
Sgpg1	TTGCAGGTTACGATAAGCGACGGGTTTTAGAGCTAGAAATAGC	sgRNA for *pgaA* knockout	2117 bp	N/A
Sgpg2	CCGTCGCTTATCGTAACCTGCAAACTAGTATTATACCTAGGAC			N/A
csF1	GCATGAGTAGAACTACTGCA	Upstream fragment for *csgD* knockout	452 bp	N/A
csF2	AGCCATGAAACGTTATTTAC			N/A
csR1	AATAACGTTTCATGGCTCTATGGACTTCATTAAACAT	Downstream fragment for *csgD* knockout	493 bp	N/A
csR2	TCGCTGATTGCTGCGATATGT			N/A
Sgcs1	TAACTATCGTTATAACAGCACGGGTTTTAGAGCTAGAAATAGC	sgRNA for *csgD* knockout	2117 bp	N/A

Sgcs2	CCGTGCTGTTATAACGATAGTTAACTAGTATTATACCTAGGAC			N/A
laF1	AAACAGGCGGCAGTAAG	Upstream fragment for *lacZ* knockout	526 bp	N/A
laF2	CGTGGTGGTTATGCCGATGACCACCAGCGAAATG			N/A
laR1	TCGGCATAACCACCACG	Downstream fragment for *lacZ* knockout	571 bp	N/A
laR2	CGCCCATCTACACCAAC			N/A
Sgla1	CATCGGGCAAATAATATCGGGTTTTAGAGCTAGAAATAGC	sgRNA for *lacZ* knockout	2117 bp	N/A
Sgla2	CCGATATTATTTGCCCGATGACTAGTATTATACCTAGGAC			N/A
Pgaar	ACGTGGCTCGATCACTTCTG	qRT-PCR of *pgaA*	205 bp	N/A
Pgaaf	CTGGTTACAGGGGCATTCGT			N/A
Csgdr	TGATGAACAACGAACGAGCG	qRT-PCR of *csgD*	264 bp	N/A
Csgdf	ACCGCGACATTGAAAACTGG			N/A
Csrar	CTGGGATTTTTCAGCCTGGATAC	qRT-PCR of *csrA*	152 bp	N/A
Csraf	GAGTTGGTGAGACCCTCATGATT			N/A
Csrbr	GCTACTTTTGTTGCTCCCTGC	qRT-PCR of *csrB*	159 bp	N/A
Csrbf	CGGTCAGGATGATTCGGTGG			N/A
Rpodr	CGCGAAGGCGAAATTGACAT	qRT-PCR of *rpoD*	224 bp	N/A
Rpodf	TGGGAAAGCTCAGAACCGAC			N/A

Supplementary Table S3. Genomic coordinates of the genes used in this study.

Gene	Genomic coordinates (CP053602.1)
rpoE	2,577,335..2,577,910
rseA	2,576,652..2,577,302
rseB	2,575,696..2,576,652
csrA	2,660,225..2,660,410
csrB	2,758,044..2,758,412
rybB	893,981..894,059
csgD	1,106,411..1,107,061
pgaA	1,095,447..1,097,870
pgaB	1,093,420..1,095,438
pgaC	1,092,102..1,093,427
pgaD	1,091,687..1,092,100
lacZ	334,068..337,138

Supplementary Table S4. Links to the software used in this study.

Software	Official website
ImageJ	https://imagej.net/ij/
fastp	https://github.com/OpenGene/fastp
STAR	https://github.com/alexdobin/STAR
StringTie	https://github.com/gpertea/stringtie
DESeq2	https://bioconductor.org/packages/release/bioc/html/DESeq2.html
SPSS 13.0	https://www.ibm.com/spss
clusterProfiler	https://bioconductor.org/packages/release/bioc/html/clusterProfiler.html
