# Supplementary material for: Magnetic Fields Disperse Escherichia coli from Biofilm via CsrA and RpoE Regulation
Source: Microorganisms. 2026 Oct 1;14(10):2200. doi: 10.3390/microorganisms14102200 (PMC13707543; doi:10.3390/microorganisms14102200)
Supplement: Supplementary file 1 [file microorganisms-14-02200-s001.zip › supplementary Methods.pdf]

1 Construction and verification of gene-overexpression strains

The gene to be overexpressed was amplified from the genomic DNA of *E. coli* BL21(DE3) by PCR using the corresponding primers. The PCR product and the pET-28a vector were digested with appropriate restriction endonucleases. The two fragments were ligated using T4 DNA ligase (Takara, Shiga, Japan), and the ligation product was transformed into BL21(DE3) competent cells using the heat-shock method.

For co-expression of two genes, a complete expression box of one gene was gained from its overexpression plasmid by PCR. Then the expression box was connected to another gene overexpression plasmid by seamless cloning (pEASY - Basic Seamless Cloning and Assembly Kit, TransGen Biotech, Shanghai, China).

2 Construction and verification of gene-knockout strains

The homologous arms were amplified using corresponding primers targeting the upstream and downstream sequences of the gene to be knocked out, and fused into a single fragment via PCR. Homologous arms for *csgD*, *lacZ* and *pgaA* were ~500 bp fragments amplified from regions 300–500 bp outside sgRNA target sites. For *rybB*, ~400 bp upstream and downstream arms were used, with the *gfp* gene inserted between them to enhance recombination efficiency. The single guide RNA (sgRNA) cassette was integrated into the pTargetF plasmid by PCR, generating the pTargetF-sgRNA construct, which was then transformed into *E. coli* DH5α (Weid, Shanghai, China). Positive clones were screened on spectinomycin agar plates, and plasmids were extracted for subsequent use. The pCas9 plasmid was transformed into *E. coli* BL21(DE3), and positive transformants were selected on kanamycin agar plates.

The BL21-pCas9 strain was cultured overnight in 5 mL liquid LB medium at 30 °C with shaking at 200 rpm. Then, 1 mL of the overnight culture was inoculated into 50 mL liquid LB medium and incubated at 30 °C, 200 rpm for 2 h. Next, 500 μL of 1 mol/L L-arabinose (Solarbio, Beijing, China) aqueous solution was added, and incubation was continued for another 1 h under the same conditions. Bacterial cells were harvested by centrifugation at 5000 rpm for 10 min at 4 °C. The supernatant was discarded, and the cell pellet was resuspended in sterile water followed by another centrifugation step. After removing the supernatant again, the pellet was washed with 10% (w/v) glycerol solution and centrifuged. This washing step was repeated once more. Finally, the cell pellet was resuspended in 1 mL of 10% (w/v) glycerol to prepare BL21-pCas9 competent cells.

The fused homologous fragment and pTargetF-sgRNA plasmid were co-transformed into the competent cells via electroporation. Transformants were grown overnight at 30 °C on LB agar plates containing both spectinomycin and kanamycin. The desired gene-knockout strains were identified by colony PCR.

3 SDS-PAGE

Overexpression strains were cultured and induced following the biofilm culture protocol described above. After induction, biofilm cells were detached by repeated vigorous pipetting, and the culture suspension was mixed thoroughly. 40 μL of the culture suspension was mixed with 10 μL of 5× protein loading buffer (Coolaber, Beijing, China), and the mixture was heated at 100 °C for 10 min. Subsequently, SDS-PAGE was performed by 20% PAGE

(GenScript, Nanjing, China). BlueRay prestained protein ladder (Genedirex, Taiwan, China) was used as the molecular weight marker.

4 Construction and verification of promoter activity reporter strains

The target promoter was amplified from the genomic DNA of *E. coli* BL21(DE3) by PCR with homologous arms for pET-28a multiple cloning site (MCS). Partial MCS fragment of pET-28a was amplified with Peo1/Peo2 primers. The two fragments were fused by overlap PCR. The fused product and pET-28a were double-digested by *Bgl*II and *Eco*R I, ligated and transformed into *E. coli* DH5α. Clones were validated by colony PCR and sequencing. The *lacZ* gene, amplified from BL21(DE3) genome, was inserted into the promoter-swapped reporter plasmid by double digestion. Then reporter plasmids were transformed into *E. coli* Δ*lac*Z strain.
